# A Comparison between the Ability of Revised Trauma Score and Kampala Trauma Score in Predicting Mortality; a Meta-Analysis

**Published:** 2019-01-15

**Authors:** Shahram Manoochehry, Masoud Vafabin, Saeid Bitaraf, Ali Amiri

**Affiliations:** 1 Trauma Research Center, Baqiyatallah University of Medical Sciences, Tehran, Iran.; 2 Shiraz University of Medical Sciences, Shiraz, Iran; 3 Department of Epidemiology and Biostatistics, Iran University of Medical Sciences, Tehran, Iran.

**Keywords:** Revised Trauma Score, Kampala Trauma Score, mortality

## Abstract

**Introduction:**

Describing injury severity in trauma patients is vital. In some recent articles the Revised Trauma Score (RTS) and Kampala Trauma Score (KTS) have been suggested as easily performed and feasible triage tools which can be used in resource-limited settings. The present meta-analysis was performed to evaluate and compare the accuracy of the RTS and KTS in predicting mortality in low-and middle income countries (LMICs).

**Methods:**

Two investigators searched the Web of Science, Embase, and Medline databases and the articles which their exact number of true-positive, true-negative, false-positive, and false-negative results could be extracted were selected. Sensitivity and subgroup analysis were performed using Stata software version 14 to determine the factor(s) affecting the accuracy of the RTS and KTS in predicting mortality and source(s) of heterogeneity.

**Results:**

The heterogeneity was high (I2 > 80%) among 11 relevant studies (total n = 20,631). While the sensitivity of the KTS (0.88) was slightly higher than RTS (0.82), the specificity, diagnostic odds ratio, negative likelihood ratio, and positive likelihood ratio of the KTS (0.73, 20, 0.16, 3.30, respectively) were lower than those of the RTS (0.91, 45, 0.20, 8.90, respectively). The area under the summary-receiver operator characteristic curve for KTS and RTS was 0.88 and 0.93, respectively.

**Conclusion:**

However, regarding accuracy and performance, RTS was better than KTS for distinguishing between mortality and survival; both of them are beneficial trauma scoring tools which can be used in LMICs. Further studies are required to specify the appropriate choice of the RTS or KTS regarding the type of injury and different conditions of the patient.

## Introduction:

Traumatic injury as one of the major causes of morbidity and mortality remains a worldwide concern. According to the World Health Organization (WHO) 16% of the global burden of disease refers to injuries (1). On the other hand, traumatic injuries are the cause of about 5.8 million deaths in the world. According to statistics 90% of these deaths happen in low-and middle income countries (LMICs) (2, 3). Furthermore, injuries disable about 78 million people and account for up to 30% of hospital admissions. The burden of injuries supposed to be much higher in LMICs. In this regard, about 88.3% of lost disability-adjusted life years (DALYs) and 87.9% of road traffic deaths is reported from these areas (4, 5).

Regarding the great trauma burden in countries with such a resource-limited setting, developing protocols for allocating resources in a strategically good way to minimizes injury-related morbidity and mortality, and maximizes patient survival seems necessary. Multiple injury scoring systems have been used in high-income countries (HICs) since 1970s for triage, injury description and outcome, and mortality prediction, such as injury severity score (ISS), revised trauma score (RTS), and the trauma and injury severity score (TRISS) (6, 7). RTS is known as the current standard physiologic scoring tool used in trauma setting and research in both HICs and LMICs, which is based on physiologic variables of systolic blood pressure (SBP), respiratory rate (RR) and a higher weight variable Glasgow coma scale (GCS), comparing to other variables (8-11). A review by O’Reilly et al. indicated that the RTS and ISS used in trauma registries in LMICs are superior to other injury scoring systems (12).

However, these scoring systems perform well in developed countries and their calculation and performance require not-available diagnostic tools in resource-limited setting and can be difficult to achieve. In 1996, the Kmapala trauma score (KTS) a simplified scoring system, was developed to specifically address this problem in resource-limited settings. It reflects SBP, RR, patient age, number of serious injuries and neurologic status (13, 14).

Some recent articles have been mentioned the KTS and RTS as easily performed and feasible triage tools suggested to be used instead of other complicated and difficult ones such as TRISS (15, 16). The mortality predictive ability of KTS and RTS have been compared in various studies in many countries with different methodologies. Different results on the comparison of the KTS and RTS power in prediction of mortality has been reported in these studies (3, 15, 17-21). In spite of the apparent lack of consistent results due to the heterogeneity among studies, a meta-analysis has not been yet performed. According to the fact that KTS was developed to specifically address the difficulty of calculation and performance of other trauma scoring systems in LMICs, we performed a systematic review and meta-analysis to show which one of KTS or RTS has better accuracy and precision in predicting mortality in LMICs. 

## Methods:

***Search Strategy and Study Selection***

Here, regardless of publication status, studies of the mortality predictive ability of the RTS and KTS were included. Two investigators (AA and ShM) carried out a systematic search using the electronic databases such as Embase, Web of Science, and Medline from their commencements until July 2018. The search strategy in Medline used the following terms: ((“Revised Trauma Score”[tiab] OR RTS[tiab] OR “Kampala Trauma Score”[tiab] OR KTS[tiab]) AND (((sensitivity[tiab] OR specificity[tiab]) OR “receiver operating characteristic curve”[tiab]) OR ROC[tiab] OR prognosis[tiab] OR “prognostic value” [tiab] OR prediction[tiab] OR “predictive value” [tiab])). The search strategy in Web of Science and Embase used the following terms: Revised Trauma Score, or RTS, or Kampala Trauma Score, or KTS; and sensitivity, or specificity, or receiver operating characteristic curve, or ROC, or prognosis, or prognostic value, or prediction, or predictive value. Furthermore, searching all potentially eligible references cited in related review and original articles were carried out manually in Google Scholar and Google search engines. If the full text was not accessible or the required details were not completely available in the full text, we tried to get the details from the authors by email. 

***Inclusion and Exclusion Criteria***

In order to include certain studies in the present study, they should have evaluated the performance of the RTS or KTS for patient mortality prediction in LMICs, and also the exact number of true-positive, true-negative, false-positive, and false-negative results could be extracted either directly or indirectly. The studies with the following criteria were excluded: did not evaluate the performance of RTS or KTS in predicting patient mortality; were not conducted in LMICs; were not English-language; and letters to editors or conference abstracts. The two reviewers who carried out the literature search also individually made decisions and selected studies regarding inclusion criteria (AA and ShM). 

***Data Extraction and Quality Assessment***

Using two reviewers (AA and ShM) the following details were extracted from included studies independently: study location, year of publication, the first author’s surname, study design, age, number of cases, proportion of male subjects, area under the receiver operator characteristic (ROC) curve (AUC), cut-off value, sensitivity, and specificity. Two reviewers (AA and ShM) individually used the Quality Assessment of Diagnostic Accuracy Studies-2 (QUADAS-2) tool which is widely used to assess the quality of systematic reviews of diagnostic studies (22). 

The QUADAS-2 is four-key domain tool: patient selection, index test, reference standard, and flow and timing. “Concerns regarding applicability” and “risk of bias” were assessed for the first three domains and all four domains, respectively (each item answered as “unclear”, “yes,” or “no”). To evaluate the “risk of bias,” a low risk of bias is defined as “yes” answer to all signaling questions in a domain. If any signaling question has been answered as “no”, it indicated a high risk of bias. “Applicability” section was judged as same as the bias section, excluding signaling questions.

***Statistical analysis***

The I2 test was used to evaluate the heterogeneity which was defined as low, moderate, and high in thresholds of 25%, 50%, and 75%, respectively. We used true-positive, false-positive, true-negative, and false-negative parameters to calculate the sensitivity, specificity, diagnostic odds ratio (DOR), AUC, positive likelihood ratio (PLR), and negative likelihood ratio (NLR). 

Based on differences in inclusion criteria and study population or type of injury, we selected and excluded specific studies, and then performed sensitivity analysis to identify if the results have been affected by these exclusions or not.

We performed subgroup analyses based on the number of patients. Regarding the distribution of sample size among studies, we categorized them into two different groups as small and big samples. Thus, studies with a sample size <1000 patients defined as small sample. The publication bias was assessed using a funnel plot.

Stata version 14 was used for all statistical analysis and a statistically significant difference was defined as a P-value <0.05.

## Results:

**Figure1:**
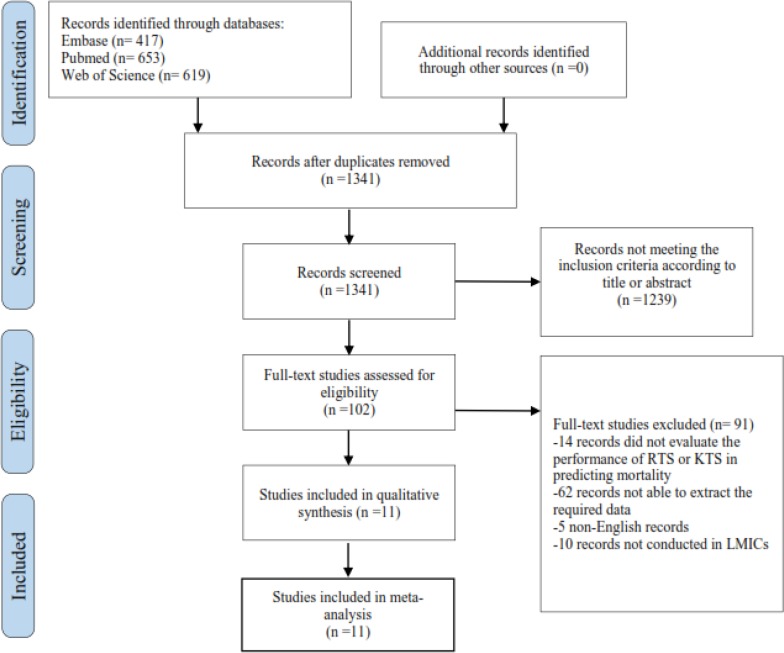
Flow diagram of systematic search for the meta-analysis considering the ability of the Revised Trauma Score and Kampala Trauma Score in predicting mortality

RTS, Revised Trauma Score; KTS, Kampala Trauma Score; LMICs, Low and Middle-Income Countries.

**Figure 2:**
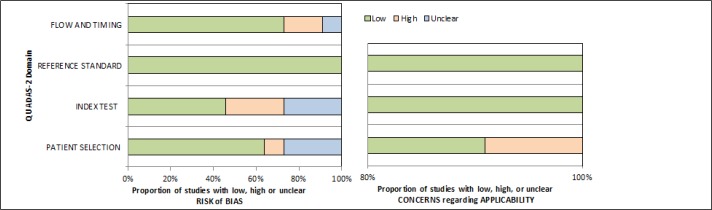
Quality assessment of included studies using QUADAS-2

The search strategy initially returned 1341 studies after removing the duplicates (Figure 1). Titles and abstracts reading led to the finding of 102 studies that used the RTS or the KTS as a prognostic method for mortality prediction. Among these 102 records, 14 articles were excluded due to not evaluating of the performance of the RTS or KTS in predicting mortality. 62 added records were excluded since the exact number of true-positive, false-positive, true-negative and false-negative test results could not be obtained, and also a total number of 5 studies that were not published in English, were excluded. An additional 10 records were excluded because they did not carry out in LMICs. Finally, after application of the mentioned-inclusion criteria, 11 eligible studies evaluating the performance of the RTS or KTS in mortality prediction in LMICs were included in this meta-analysis (Table 1) (11, 15, 16, 21, 23-29). The quality assessment of the included 11 studies are shown in Table 2 and Figure 2. The studies were conducted in six different countries, including Colombia, India, Iran, Nigeria, Turkey and Uganda. A total of 20,631 patients (ranged from 100 to 7211) were evaluated. The majority of the sample consisted of men (76.68%). Seven of 11 studies assessed only the RTS (11, 23-25, 27-29), one study assessed only the KTS (26), and three of the studies assessed both the RTS and KTS (15, 16, 21). While one study was based in a level I trauma center, other studies included patients from hospitals (28). The mortality rate and cut-off point (a threshold for mortality prediction) were different among these studies. If the exact number of all true-positive, false-positive, true-negative and false-negative test results were not directly obtained from the studies, they have been calculated indirectly from the number of patients, mortality values, sensitivity and specificity.

Using a random-effects model, we pooled related statistical parameters owing to the high level of heterogeneity (I > 80%). For the RTS, the pooled estimates were 0.82 (95% CI: 0.66–0.92) for sensitivity, 0.91 (95% CI: 0.81–0.96) for specificity, 8.90 (95% CI: 4.50–17.80) for PLR, 0.20 (95% CI: 0.10–0.39) for NLR, 45 (95% CI: 21–99) for DOR. For the KTS, the pooled estimates were 0.88 (95% CI: 0.70–0.96) for sensitivity, 0.73 (95% CI: 0.57–0.85) for specificity, 3.30 (95% CI: 2.00–5.60) for PLR, 0.16 (95% CI: 0.06–0.44) for NLR, 20 (95% CI: 6–69) for DOR (Table 3). 

**Figure 3:**
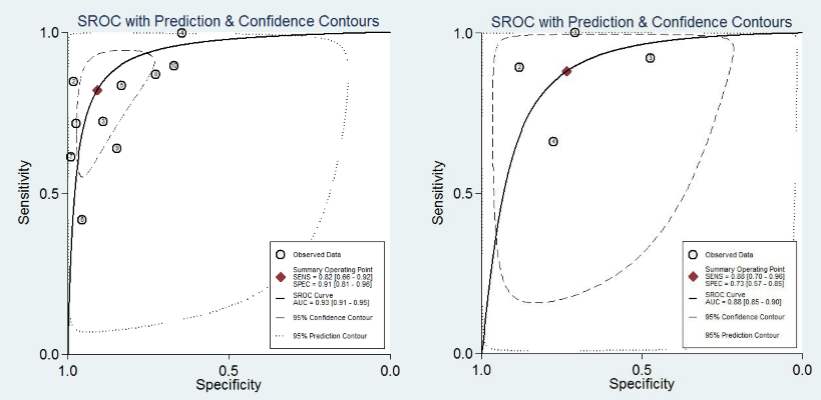
Summary receiver operating characteristic curves (SROC) of Revised Trauma Score (left) and Kampala Trauma Score (right). AUC, area under the receiver operating characteristic curve

Figure 3 shows the summary receiver operator characteristic curves (SROC) for the RTS and KTS plots. The AUC for the RTS was higher comparing to that for the KTS (0.93 vs 0.88, respectively).

In order to determine the source of heterogeneity, sensitivity analysis was conducted (Table 4). The studies by Yousefzadeh-Chabok et al. and Nakhjavan-Shahraki et al. were removed because only elders and children samples were included, respectively (23, 29). The studies by Senturk et al. and Macleod et al. were removed because they included only multiple blunt and major trauma patients, respectively (15, 25). The study by Valderrama-Molina et al. was removed because it was the only study conducted in a fourth level hospital which is equivalent to a level one trauma center in the USA (28). The I^2^ value for sensitivity did not change, and the sensitivity value was similar to that found without excluding mentioned studies.

**Figure 4:**
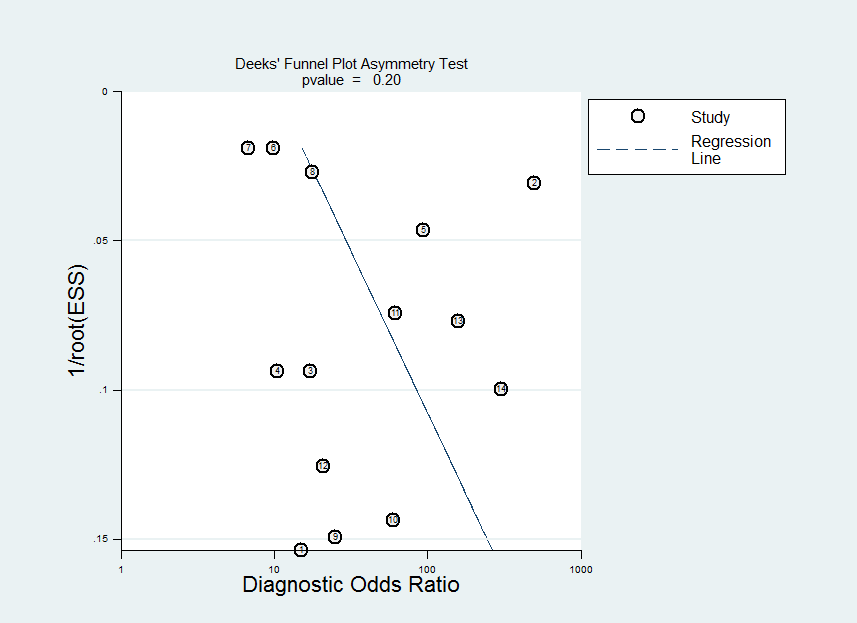
The funnel plot for assessment of publication bias

**Table 1:** General characteristics of the included studies

First Author	Country	Sample size	Mortality	Year	Age (Years)	Male (%)	Tool	Cut-Off value	TP	FP	FN	TN	AUC	Sen (%)	Spe (%)
Ahun	Turkey	100	0.120	2014	18	77	RTS	5.97	5	4	7	84	0.68	41.67	95.45
Eftekhar	Iran	7211	0.038	2005	Mean, 32.5	76	RTS	4.09*	273	2463	1	4474	0.91	99.80	64.50
Macleod	Uganda	150	0.255	2003	15	89.3	RTS KTS	7.40 13.0	34 35	37 59	4 3	75 53	0.87 0.84	89.5 92.1	67.0 47.3
Nakhjavan-Shahraki	Iran	2184	0.057	2017	18	75.56	RTS	1.0	88	53	35	1972	0.86	71.54	97.38
Nakhjavan-Shahraki	Iran	814	0.031	2017	18	74.32	RTS	1.0	22	14	4	774	0.94	84.6	98.2
Oluwadiya	Nigeria	186	0.065	2010	Mean, 36.7	73.1	RTS KTS	5.7 12.0	10 12	29 51	2 0	145 123	0.88 0.91	83.3 100	83.3 70.7
Owor	Uganda	1305	0.036	2001	.	.	KTS	12.0	42	151	5	1107	0.87	90.0	88.0
Roy	India	4091	0.22	2016	Mean, 36.5	83	RTS KTS	7.0 12.0	574 595	486 716	326 305	2705 2475	0.81 0.74	63.8 66.07	84.78 77.55
Senturk	Turkey	153	0.118	2013	18	81	RTS	4.08	13	15	5	120	0.80	72.0	89.0
Valderrama-Molina	Colombia	4085	0.093	2016	15	84.2	RTS	6.37	330	1010	50	2695	0.86	86.77	72.75
Yousefzadeh-Chabok	Iran	352	0.139	2016	Mean, 71.5	53.4	RTS	6.0	30	3	19	300	0.87	62.0	99.0

RTS, Revised Trauma Score; KTS, Kampala Trauma Score; TP, true-positive; FP, false-positive; FN, false-negative; TN, true-negative; Sen, sensitivity; Spe, specificity; AUC, area under the receiver operator characteristic curve.

* Because the cut-off value could not be extracted from the study, we set the standard cut-off value as said by Champion et al (6, 7).

**Table 2:** Quality assessment of included studies using QUADAS-2

**Study**	**Risk Of Bias**				**Applicability Concerns**		
	Patient Selection	Index Test	Reference Standard	Flow And Timing	Patient Selection	Index Test	Reference Standard
**Ahun **	1?	▲	●	●	●	●	●
**Eftekhar**	●	●	●	●	●	●	●
**Macleod**	●	▲	●	▲	●	●	●
**Nakhjavan-Shahraki**	●	●	●	●	●	●	●
**Nakhjavan-Shahraki**	?	●	●	●	●	●	●
**Oluwadiya**	●	2?	●	3?	●	●	●
**Owor**	▲	?	●	●	▲	●	●
**Roy**	●	▲	●	●	●	●	●
**Senturk**	●	●	●	●	●	●	●
**Valderrama-Molina**	●	?	●	●	●	●	●
**Yousefzadeh-Chabok**	?	▲	●	●	●	●	●

●Low Risk ▲High Risk ? Unclear Risk

1: The sampling technique (consecutive or random) is undisclosed, 2: the interpretation of index test results without knowledge of the results of the reference standard was unknown or pre-specification of threshold was unclear, 3: the proportion of study population, in which included in analysis is unknown. QUADAS-2 Quality Assessment of Diagnostic Accuracy Studies 2.

**Table 3:** Pooled estimates of the Revised Trauma Score and Kampala Trauma Score

**Tool**	**Sensitivity **	**Specificity**	**PLR**	**NLR **	**DOR**
**RTS**	0.82 (0.66-0.92)	0.91 (0.81-0.96)	8.9 (4.5-17.8)	0.20 (0.10-0.39)	45 (21-99)
**KTS**	0.88 (0.70-0.96)	0.73 (0.57-0.85)	3.3 (2-5.6)	0.16 (0.06-0.44)	20 (6-69)

All measures were presented with 95% confidence interval. RTS, Revised Trauma Score; KTS, Kampala Trauma Score; CI, confidence interval; PLR, positive likelihood ratio; NLR, negative likelihood ratio; DOR, diagnostic odds ratio.

**Table 4:** Sensitivity analysis results

**First Author**	**Sensitivity (95%CI)**	**Specificity (95%CI)**	**AUC**	**I2(%)**
**None** *	0.82 (0.66-0.92)	0.91 (0.81-0.96)	0.93	100
**Macleod**	0.81 (0.62-0.92)	0.92 (0.83-0.97)	0.94	100
**Nakhjavan-Shahraki**	0.82 (0.63-0.92)	0.89 (0.78-0.95)	0.92	100
**Senturk**	0.83 (0.65-0.93)	0.91 (0.80-0.96)	0.94	100
**Valderrama-Molina**	0.82 (0.63-0.92)	0.92 (0.82-0.97)	0.94	100
**Yousefzadeh-Chabok**	0.84 (0.67-0.93)	0.89 (0.78-0.94)	0.93	100

* No article was excluded. CI, Confidence Interval; AUC, area under the receiver operator characteristic curve.

**Table 5:** Subgroup meta-analysis for the Revised Trauma Score

**Subgroup**	**Sensitivity (95%CI)**	**Specificity (95%CI)**	**AUC**	**I2(%)**
**All**	0.82 (0.66-0.92)	0.91 (0.81-0.96)	0.93	100
**Sample size 1000**	0.76 (0.62-0.86)	0.94 (0.83-0.98)	0.89	97
**Sample size 1000**	0.89 (0.57-0.98)	0.85 (0.64-0.95)	0.93	100

CI, Confidence Interval; AUC, area under the receiver operator characteristic curve.

Subgroup meta-analysis was performed by classifying studies according to their number of samples. The changes in sensitivity and specificity values are shown in Table 5. Due to the low number of studies, we could not perform this analysis in detail for KTS. Therefore, only subgroup meta-analysis for RTS was performed. 

In order to explore publication bias (which obtained P-value of 0.2 did not suggest it), a funnel plot asymmetry analysis was used (figure 4).

## Discussion

Although the KTS was developed to simplify the mortality prediction in LMICs, the present meta-analysis showed high accuracy of the KTS and RTS in predicting mortality with a slightly better performance for RTS. The specificity, DOR, NLR, and PLR of the KTS were slightly lower than those of the RTS, while the sensitivity of the KTS was slightly higher than RTS.

In emergency settings, physicians are always challenging with the issue of classifying the trauma patients according to the severity of their trauma (24). In this regard, different trauma scoring systems are frequently used for diagnosing high risk patients. However, each one of them has its own advantages and disadvantages. Trauma related deaths are classified into three different groups. Group one (50%) consists of patients who die at the scene (often because of severe vascular injury or major head trauma). Group two (30%) includes patients with hospital admission who die within the first hours of admission called “golden hour”. The deaths of this group are usually because of major head, thorax or abdominal trauma. Group three (20%) includes those who die at a later time (usually due to multi-organ failure or sepsis). The mortality rate of two later groups can be decreased through fast and efficient treatment approaches (26, 27, 30, 31). Therefore, a precise selection of trauma scoring system especially in LMICs with a much higher rate of trauma is an essential issue.

However, the RTS necessitates the use of a formula to admit the GCS, respiratory rate and systolic blood pressure and it remains the most commonly used pre-hospital trauma scoring system. Moreover, the RTS is considered as one of the most easily calculated triage tools available shortly after trauma admission (25, 32, 33). Like the RTS, the KTS is an easily performed trauma scoring system without specific requirements such as finance resource, the associated manpower need and retrospective review of injuries. Therefore, it is a reasonable and feasible choice for front-line triage usage (15). Some recent articles have been mentioned the KTS and RTS as easily performed and feasible triage tools suggested to be used instead of other complicated tools such as TRISS (15, 16). Different results on the comparison of the mortality predictive ability of KTS and RTS has been reported. Despite the apparent lack of consistent results due to the heterogeneity among studies, a meta-analysis has not been yet performed to compare the accuracy of RTS and KTS. According to the fact that KTS was developed to specifically address the difficulty of calculation and performance of other trauma scoring systems in LMICs, we performed a systematic review and meta-analysis to show which one of KTS or RTS has better accuracy and precision in predicting mortality in LMICs.

Stability of sensitivity and specificity can be integrated to make a comprehensive test performance named likelihood ratios, which is superior to their components. The PLR of the RTS (8.90) was higher than that of the KTS (3.30), with a positive RTS or KTS outcome indicating an approximately nine and three-fold higher risk of mortality, respectively. However, it should be considered that PLR should be more than 10 so as to be useful. The slightly higher NLR for the RTS (0.20) suggests that it had a slightly lower accuracy comparing to the KTS (0.16) for predicting survival.

The DOR combines the sensitivity and specificity values into a single number indicating the test accuracy (between zero and infinity). Higher DOR values indicate higher accuracy of a test (better discriminatory performance), and a DOR value of 1.0 indicates that the RTS or KTS does not distinguish between mortality and survival. In the present study, the higher DOR of the RTS (DOR = 45) indicates that it is more capable than the KTS (DOR = 20) in distinguishing between mortality and survival.

In addition to summarizing the sensitivity and specificity, the SROC curve is a more effective index than the other indices (since the threshold effect has no influence on it). Based on the SROC curve in our study, the AUC of the RTS (0.93) was slightly higher than that of the KTS (0.88).

All of these indices indicate that the overall accuracy of the RTS and KTS in predicting mortality was as high as expected, and both of them had high sensitivity and specificity. In the three studies evaluating the RTS and KTS, two studies (16, 21) concluded that the accuracy of the RTS and KTS for predicting mortality was similar, while another study showed that the RTS is superior to the KTS (15). Based on the obtained results, we found that the RTS has slightly higher accuracy than the KTS in predicting mortality.

The accuracy of RTS in predicting mortality has been affected by some factors and was different in each subgroup. The sensitivity value of RTS was higher with sample size ≥1000, indicating better result of mortality prediction in studies with a higher sample size. Moreover, the sensitivity was not changed by removing the study of Valderrama-Molina et al. (which was the only study conducted in a level one trauma center) indicating no difference of the RTS performance in a hospital or a level one trauma center. The sensitivity value of RTS increased after removing the study by Yousefzade-Chabok et al., which shows worse performance of RTS in elder populations.

After removing the specific studies, the heterogeneity did not change. The subgroup meta-analysis showed that the differences in the number of patients between studies contributed to the heterogeneity. Thus, studies with a larger sample size contributed more to the heterogeneity. The differences in the populations and countries can also effect on the heterogeneity.

The present study had several limitations. First, we used only English written studies. Second, due to the lack of data, we focused only on the ability of mortality prediction and excluded physiology and age that could influence the trauma outcome. Third, because of the limited data, we could not analyze additional factors such as type of injury, age, ethnic group and treatment which might change the RTS or KTS accuracy. To the best of our knowledge, this is the first study on the ability of the RTS and KTS in mortality prediction as a meta-analysis.

## Conclusions

Briefly, high accuracy of both the RTS and KTS for predicting mortality in LMICs has been confirmed. However, regarding accuracy and performance, the RTS was better than the KTS for distinguishing between mortality and survival; both of them are beneficial trauma scoring tools which can be used in resource-limited settings. Further studies are required to specify the appropriate choice of the RTS or KTS regarding the type of injury and different conditions of the patient.
